# Dietary insulinemic potential, sleep quality and quantity in Iranian adults: Yazd health study and TAMYZ study

**DOI:** 10.1186/s40795-023-00745-6

**Published:** 2023-07-25

**Authors:** Peyman Sarsangi, Mohammad Mohammadi, Amin Salehi-Abargouei, Ahmad Esmaillzadeh, Masoud Mirzaei

**Affiliations:** 1 Department of Community Nutrition, School of Nutritional Sciences and Dietetics, Tehran University of Medical Sciences, Tehran, Iran; 2 Department of Community Medicine, School of Medicine, Hormozgan University of Medical Sciences, Bandar Abbas, Iran; 3 Research Center for Food Hygiene and Safety, School of Public Health, Shahid Sadoughi University of Medical Sciences, Yazd, Iran; 4 Department of Nutrition, School of Public Health, Shahid Sadoughi University of Medical Sciences, Yazd, Iran; 5 Yazd Cardiovascular Research Center, Non-Communicable Diseases Research Institute, Shahid Sadoughi University of Medical Sciences, Yazd, Iran; 6 Obesity and Eating Habits Research Center, Endocrinology and Metabolism Molecular Cellular Sciences Institute, Tehran University of Medical Sciences, Tehran, Iran; 7 Food Security Research Center, Department of Community Nutrition, Isfahan University of Medical Sciences, Isfahan, Iran

**Keywords:** Sleep duration, Sleep quality, Insulin load, Insulin index, Adults, Cross-sectional

## Abstract

**Background & aims:**

To examine the link between dietary insulin index (DII) and load (DIL) and sleep duration/quality for the first time.

**Methods:**

This cross-sectional study conducted on data from the recruitment phase of Yazd Health Study (YaHS)-Yazd Nutrition Study (TAMYZ), prospective study in Yazd, central Iran. Data on demographic characteristics, dietary intakes, sleep quantity and quality, and potential confounders were gathered by interview. Sleep quality and its components (insufficient sleep, delay in falling asleep, medication use for sleep, and sleep disorder) were assessed by a modified version of Pittsburgh questionnaire. The link between DII/DIL and low sleep quality and short/long sleep duration was studied using multivariable logistic regression.

**Results:**

In total, 5925 individuals aged 20 to 70 were eligible to take part in the current study. After adjustment for all potential confounders, participants in the highest DIL score tertile had a lower chance for sleep disorder (OR = 0.38; 95%CI: 0.17–0.85, P_trend_ = 0.02) and delay in falling asleep (OR = 0.66; 95%CI: 0.42–1.03, P_trend_ = 0.05) compared to those in the lowest tertile. The DII was also linked to a lower chance for sleep disorder (OR = 0.61; 95% CI: 0.39–0.93, P trend = 0.02). The DIL was inversely associated with sleep medication use and delay in falling sleep in men and women, respectively (*P* < 0.05). Moreover, DII was linked to a decreased odds of sleep disorder in women (*P* < 0.05). The associations were observed in those with overweight or obesity but not in those without overweight (*P* < 0.05).

**Conclusion:**

Higher DIL and DII might be associated with sleep quality and its components. Prospective investigations are needed in the future to confirm these findings.

**Supplementary Information:**

The online version contains supplementary material available at 10.1186/s40795-023-00745-6.

## Introduction

Sleep is vital to human health, as insufficient sleep duration and/or quality is considered a serious public health problem, worldwide [1]. A study in 12 US states found that 35.3% of participants slept less than 7h a day, on average [2].Obesity [3], diabetes [4–6], cardiovascular disease [7, 8], cancer [4, 5, 9], depression [10] and mortality [11, 12] have all been linked to insufficient sleep quantity and quality.

Sleep quality and duration have been proven to be influenced by dietary intakes [13]. Consumption of milk and dairy products [14] fruit and vegetables [15] was associated with improved sleep quality. Dietary carbohydrates have also been examined to affect sleep time and quality [16]. Carbohydrates increase plasma tryptophan concentrations [17], which serves as a precursor for brain serotonin, a sleep-inducing agent [18]. Not only the quantity of carbohydrates, but also its quality is regarded important in influencing sleep quality. Dietary glycemic load and its insulinemic potential may also be important [17, 19–21]. For instance, consumption of high GI foods reduced sleep onset latency [17], increased sleep duration [19], and promoted usual sleep quality [20]. Dietary insulin index and dietary insulin load have recently been proposed as ways to measure postprandial insulin responses to all dietary components, including amino acids and lipids. These indices are determined by adding the product of the food insulin index, the energy content, and the frequency with which food items are consumed. They are thought to be a better predictor of insulin demand than the dietary glycemic index and dietary glycemic load [22].

In Iran, determining the relationship between dietary insulin index and dietary insulin load and sleep length and quality is very important, where people are eating high amounts of carbohydrates) roughly 60% of total caloric intake) mostly in the form of refined grains in their diet [23, 24]. Furthermore, a high prevalence of low sleep duration and quality are reported in Iranians [25]. To the best of our knowledge, there was no previous study on the contribution of dietary insulin index and dietary insulin load to sleep duration/quality, particularly in the Middle East.

The current study aimed to examine the association between dietary insulin index and dietary insulin load with sleep quality and quantity among a large sample of Iranian adults living in the center of the country.

## Participants and methods

### Study design and population

The abstract of this study has already been presented in a congress [26].The current cross-sectional analysis was conducted on the data from recruitment phase of Yazd Health Study (YaHS), a prospective cohort study on 9962 adults’ aged 20 to 70 residents in Yazd, Iran, between 2014 and 2016. Assessment of dietary intakes of these individuals was done in another study named Taghzieh Mardom Yazd (TAMYZ), which we used its data in the current analysis too. Details about YaHS-TAMYZ studies have already been published [27]. All participants in the YaHS-TAMYZ study signed informed consent In the current analysis, during the screening process, participants were excluded if they had missing data on sleep quantity or had missed responding more than 70 items of dietary intake data (*n* = 850) or unexplained energy intake (< 800 kcal/d or > 6000kcal/d) (*n* = 864), reported less than 3h or more than 12h of sleep per night (*n* = 250), were pregnant (*n* = 104) and reported to have major depression (*n* = 91). Those who had a history of chronic conditions at the time of the baseline survey, such as diabetes, cancer, or cardiovascular disease, were also eliminated (*n* = 1908).

### Dietary intake assessment

Participants in the YaHS study had their dietary intakes assessed separately using a semi-quantitative food frequency questionnaire (FFQ) consisting of 178 food items, whose validity and reliability had been measured and confirmed in a prior study [28]. The FFQ used in this study was a 178 item questionnaire that contained 168 food items commonly consumed in Iran and 10 questions related to the consumption of traditional foods in Yazd. Participants were asked to respond to a 10-choice frequency response section for each food item, ranging from "never or less than once a month" to "10 or more times per day" for each food item. For each food item, five different portion sizes were asked based on Iranian’s standard serving size. Participants had to answer two questions in this questionnaire: the frequency of food consumption and the amount of food consumed at each time of consumption. Using household measures, the quantity and frequency of daily intake of all food items were converted to grams per day [29]. United States Department of Agriculture's (USDA) food composition database was used to calculate each participant's energy and nutrient intakes [30].

### Calculation of dietary insulin index and load

The dietary insulin index was computed using previously published estimations by Brand-Miller et al., which are based on the insulin index of dietary items [31, 32]. The food insulin index (FII) is the difference between the area under the curve after ingestion of a 1000-kJ portion of the test food and the area under the curve after ingestion of a 1000-kJ portion of the reference food during a 2-h period. The FII of similar food items based on the similarity between their energy, carbohydrate, fiber, fat, and protein content was applied in the current investigation for food items that were not available in the food list released by Brand-Miller et al. (Supplementary Table 1). First, the insulin load of each food was determined using the following formula to determine dietary insulin load (DIL):

$$Insulin\,load\,of\,a\,given\,food=Insulin\,index\,of\,that\,food \times\,energy\,content\,of\,that\,food\,(kcal/d)$$

DIL was calculated for each subject by adding up the insulin load of consumed foods. The dietary insulin index (DII) was then computed for each participant by dividing DIL by total calorie intake.

### Assessment of sleep quantity and quality

Data on regular sleep quantity was collected by an interview utilizing a nocturnal sleep questionnaire in the YaHS study. Sleep duration was divided into three categories: fewer than 5 h (short sleep duration), 5 to 8 h (normal sleep duration), and more than 8 h (long sleep duration) [33, 34]. The sleep quality of the study participants was obtained through an abbreviated form of the Pittsburgh questionnaire. The validity and reliability of the complete Pittsburgh questionnaire for the Iranian population has been confirmed [35]. The sleep quality score in the complete Pittsburgh questionnaire is between 0 and 21. In the present study, due to the use of the abbreviated form of the Pittsburgh questionnaire, the average sleep quality score was between 0 and 11, which was calculated based on the following questions: 1. How many minutes does it take to fall asleep from the time you go to bed? [Less than 15 min (0 score), 16–30 min (1 score), 31–60 min (2 scores), more than 1 h (3 scores)]. 2. How many times in the last 30 days have you been unable to fall asleep within half an hour?[None (0 points), less than once per week (1 point), once or twice per week (2 points), three or more times per week (3 points)]. The sum of these two questions, which is a number between 0 and 6, is known as the delay in falling asleep, and the delay in failing asleep was categorized as follows: score 0 (got 0), score 1 to 2 (got 1), score 3 to 4 (got 2), score 5 to 6 (got 3). 3. How many hours a night do you sleep? [More than 7 h (0 score), 6 to 7 h (1 score), 5 to 6 h (2 scores) and less than 5 h (3 scores)]. 4. How many times in the past month have you taken sleeping pills or sedatives to fall asleep? [None (0 score), once a week (1 score), twice a week (2 scores), three or more times a week (3 scores)]. 5. Did you wake up in the middle of the night or early in the morning? [None (0 score), less than once a week (1 score), once or twice a week (2 scores), three or more times a week (3 scores)] 6. How many times did you have nightmares while sleeping? [None (0 score), less than once a week (1 score), once or twice a week (2 scores), three or more times a week (3 scores)] 7. How many times did you wake up in the middle of the night to go to the bathroom? [None (0 score), less than once a week (1 score), once or twice a week (2 scores), three or more times a week (3 scores)]. Answers to questions 2 and 4 to 7, is known as sleep disorder, were added and converted as follows: score zero (0), score 1 to 9 (score 1), for score 10 to 12 (score 2). Finally, the sleep quality score in this study was generated by adding the scores of the above-mentioned questions and was ranged from 0 to 11 [delay in falling asleep (0–3), night sleep (0–3), sleep medication use (0–3), sleep disorder (0–2)].

### Anthropometric measurements

Anthropometric markers such as height and weight were objectively measured in the YaHS-TAMYS study with minimum outfits and no shoes. Body weight was measured to the nearest 0.1 kg using a digital scale and body analyzer (Omron BF511, Omron Inc. Nagoya, Japan). Using a tape measure on a straight wall, height was measured in standing posture to the closest centimeter. The formula for calculating the body mass index (kg/m^2^) was: weight (kg) divided by height squared (m^2^).

### Assessment of other variables

Age, sex, physical activity expressed as metabolic equivalent hours per week (MET-h/wk), job, BMI, smoking status, education level, marital status, and average duration of watching television and mobile use per day were all collected using predefined questionnaires by professional interviewers and were considered possible confounders. The short form of the standard physical activity questionnaire (IPAQ), which is developed to track the level of physical activity in developing countries, was used to assess physical activity [36]. The abbreviated form of physical activity includes questions related to four levels of physical activity such as intense, moderate, walking, and sitting activities over the past seven days, the intensity, duration, and repetition of which are completed by individuals. In this questionnaire, intensive physical activity is defined as at least a 10-min exercise that generates a significant rise in respiration, heart rate, and sweating. Also, moderate activity in this questionnaire is an activity that has a minimum duration of 10 min and increases the average respiration, heart rate, and sweating. This questionnaire is standard and its reliability and validity have been confirmed [37].

### Statistical analysis

Subjects were categorized into tertiles based on their total DII and DIL. For continuous data, mean ± standard error (SE) was used, and for categorical variables, percentage was used. The one-way analysis of variance (ANOVA) and chi-square test were used to examine the difference in quantitative and qualitative variables between tertiles of dietary DII/DIL. The analysis of covariance (ANCOVA) was used to compare dietary nutrient intake between the tertiles of DII/DIL, with adjustments for sex, age and energy intake. Logistic regression in crude and multi-variable adjusted models was done to assess the association between DII or DIL and the chance for developing short (< 5 h) vs. normal (5–8 h), long (> 8 h) vs. normal (5–8 h) sleep duration, and insufficient sleep (< 5 h) vs. sufficient sleep (≥ 5 h), low sleep quality (total score 8 and more) vs. normal sleep quality (total score 7 and less), delay in falling asleep (delay in falling asleep score 3) vs. low delay in falling asleep (delay in falling asleep score 2 and less), using medication for sleep [sleep medication use three or more times a week (score 3)] vs. low medication use for sleep [sleep medication use two or less times a week (score 2 or less)], have sleep disorder (sleep disorder score 2) vs. no sleep disorder (sleep disorder score 1 or less). The first tertiles of DII and DIL were used as a reference group to calculate odds ratios (ORs) and 95% confidence intervals (CIs). The first model was adjusted for age, gender, and total calorie intake. In model 2, additional adjustments were performed for BMI (continuous), education (categorizing into Primary school and less/High school/ Diploma and Graduate Diploma/ Bachelor student/ Master student and Doctorate), physical activity (continuous), marital status (single/ married/ widowed or divorced), smoking status (never smoker/ current smoker/ ex-smoker) job situation (categorizing into unemployed/ government employee/ manual worker/ self-employed), anxiety (continuous), stress (continuous), caffeine (continuous), duration of cellphone use (continuous) and watching television & movie (continuous). In addition, in model 3, additional adjustments were made for the usage of sleeping medications (more than twice a week). SPSS software was used for all statistical analyses (version 26.0; SPSS Inc, Chicago IL). Statistical significance was defined as a *P* value < 0.05.

## Results

This study included a total of 5925 participants, ranging in age from 20 to 70 years old (3035 males and 2890 females). Table 1 shows the overall characteristics of participants across dietary DII and DIL tertiles. Participants in the top tertile of DII were more likely to be married, current smoker, and were less likely to be educated, physically active, have stress, and use cellphone compared with those in the bottom tertile (*P* < 0.05). Moreover, participants in the highest tertile of DIL were more likely to be male, physically active, and use cellphone compared with those in the lowest tertile (*P* < 0.05). Also mean BMI and distribution of participants in terms of age categories, educated, job status, marital status (married) were significantly different across DIL tertiles (*P* < 0.05 for all).

**Table 1:** General characteristics of participants across tertiles of dietary insulin index and dietary insulin load^1^

Variables	Dietary insulin index			P^2^	Dietary insulin load			P^2^
	T_1_	T_2_	T_3_		T_1_	T_2_	T_3_	
**Subjects, n**	1976	1977	1976		1976	1977	1976	
**Sex (male, %)**	51.3	49.9	52.5	0.266	47.5	51.8	54.3	< 0.001
**Age (year, %)**				0.010				< 0.001
20–29	25.8	23.0	22.5		20.9	25.4	25.0	
30–39	23.0	23.9	23.6		20.6	24.5	25.3	
40–49	23.4	23.5	21.3		24.1	22.1	21.9	
50–59	16.7	15.9	18.4		18.3	16.3	16.5	
60–69	11.1	13.7	14.2		16.1	11.7	11.3	
**Education (Diploma and Graduate Diploma, %)**	35.4	31.0	33.0	0.007	31.7	33.8	34.0	< 0.001
**Job Status, %**				0.223				0.001
Unemployed	19.3	18.6	21.4		20.0	19.3	20.1	
Government employee	47.0	48.4	45.3		50.4	46.9	43.4	
Manual worker	4.0	3.4	3.2		3.6	3.3	3.7	
Self-employed	29.5	29.6	30.1		26.0	30.5	32.8	
**Married, %**	83.0	84.7	85.8	0.017	84.9	83.6	84.8	< 0.001
**Smoking status (Current smoker, %)**	10.5	10.2	10.9	0.045	10.5	10.6	10.5	0.795
**BMI (kg/m**^**2**^**)**	26.6 ± 0.1	26.6 ± 0.1	26.6 ± 0.1	0.936	26.8 ± 0.1	26.4 ± 0.1	26.6 ± 0.1	0.027
Physic activity (MET*min/wk)	948 ± 21	855 ± 19	916 ± 21	0.006	870 ± 20	894 ± 20	956 ± 21	0.010
**Cell phone use (min/day)**	30.4 ± 1.2	26.8 ± 1.2	25.6 ± 1.1	0.015	24.0 ± 1.1	29.5 ± 1.2	29.4 ± 1.2	0.001
**Watching television & movie (h/day)**	3.74 ± 0.05	3.77 ± 0.05	3.83 ± 0.05	0.528	3.78 ± 0.05	3.76 ± 0.05	3.80 ± 0.05	0.923
**Anxiety (score)**	2.63 ± 0.07	2.57 ± 0.07	2.75 ± 0.07	0.199	2.74 ± 0.07	2.70 ± 0.07	2.51 ± 0.07	0.083
**Stress (score)**	5.83 ± 0.10	5.44 ± 0.10	5.54 ± 0.10	0.019	5.55 ± 0.10	5.54 ± 0.10	5.72 ± 0.10	0.394

*PA* physical activity, *ME* metabolic equivalent, *BMI* body mass index;

^1^Values are mean ± SE otherwise indicated

^2^P are resulted from chi-square for qualitative variables and from ANOVA for quantitative variables and from

Age, sex, and energy adjusted dietary intakes of selected foods and nutrients across tertiles of DII and DIL are shown in Table 2. Participants in the highest tertile of DII had a higher intake of total carbohydrates, thiamine, whole grains, refined grains, fruits, caffeine, and a lower intake of total energy, total fat, saturated fat, monounsaturated fat, poly-unsaturated fat, total protein, simple sugar, magnesium, vitamin E, vitamin B6, vitamin B12, folic acid, calcium, zinc, riboflavin, high-fat dairy products, low-fat dairy products, processed meats, red meats, nuts, legumes, vegetables compared with those in the lowest tertile (*P* < 0.05). Higher DIL was associated with greater intakes of total energy, total protein, total carbohydrate, vitamin C, thiamine, riboflavin, calcium, iron, zinc, whole grains, refined grains, legumes, red meats, processed meats, fruits, vegetables and, a lower intake of total fat, saturated fat, monounsaturated fat, poly-unsaturated fat, vitamin E, B6, folic acid, nuts (*P* < 0.05).

**Table 2:** Comparison of age, gender and energy adjusted dietary food groups and nutrients intake according to tertiles of dietary insulin index and dietary insulin load

	Dietary insulin index				Dietary insulin load			
	T1	T2	T3	*P*-Value	T1	T2	T3	*P*-Value
**Total energy (Kcal/day)**	3126.8 ± 25.8^a^	2660.5 ± 25.8^b^	2532.5 ± 25.8^c^	< 0.001	1699.0 ± 13.9^a^	2509.1 ± 13.8^b^	4111.8 ± 13.8^c^	< 0.001
**Nutrients**^**1**^								
Total fat (g/day)	122.0 ± 0.7^a^	104.0 ± 0.7^b^	92.1 ± 0.7^c^	< 0.001	125.1 ± 1.0^a^	110.0 ± 0.7^b^	83.0 ± 1.1^c^	< 0.001
Saturated fat (g/day)	33.7 ± 0.2^a^	29.0 ± 0.2^b^	26.3 ± 0.2^c^	< 0.001	32.3 ± 0.3^a^	30.6 ± 0.2^b^	26.1 ± 0.3^c^	< 0.001
Mono-unsaturated fat (g/day)	37.8 ± 0.3^a^	31.7 ± 0.2^b^	27.9 ± 0.2^c^	< 0.001	38.7 ± 0.4^a^	34.1 ± 0.3^b^	24.8 ± 0.4^c^	< 0.001
Poly-unsaturated fat (g/day)	32.8 ± 0.3^a^	26.2 ± 0.3^b^	23.0 ± 0.3^c^	< 0.001	33.5 ± 0.5^a^	29.2 ± 0.3^b^	19.3 ± 0.6^c^	< 0.001
Total protein (g/day)	118.9 ± 0.8^a^	109.2 ± 0.7^b^	101.6 ± 0.7^c^	< 0.001	108.7 ± 1.1^a^	111.9 ± 0.8^b^	109.0 ± 1.2^ab^	0.007
Total carbohydrate (g/day)	340.3 ± 1.6^a^	397.6 ± 1.6^b^	429.3 ± 1.6^c^	< 0.001	360.1 ± 2.5^a^	375.0 ± 1.8^b^	432.0 ± 2.8^c^	< 0.001
Simple sugar (g/day)	299.7 ± 10.1^a^	272.3 ± 9.9^ab^	245.7 ± 10.0^b^	0.001	286.1 ± 14.1	268.0 ± 10.2	263.6 ± 16.0	0.521
Vitamin C (µm /d)	200.8 ± 3.4	207.9 ± 3.4	201.4 ± 3.4	0.264	176.9 ± 4.8^a^	20.93 ± 3.5^b^	225.9 ± 5.4^c^	< 0.001
Vitamin E (mg /d)	13.8 ± 0.2^a^	10.7 ± 0.2^b^	8.9 ± 0.2^c^	< 0.001	12.3 ± 0.3^a^	11.8 ± 0.2^a^	9.2 ± 0.3^b^	< 0.001
Thiamine (mg/d)	2.05 ± 0.01^a^	2.20 ± 0.01^b^	2.28 ± 0.01^c^	< 0.001	1.95 ± 0.02^a^	2.19 ± 0.01^b^	2.38 ± 0.02^c^	< 0.001
Riboflavin (µm/d)	2.38 ± 0.01^a^	2.31 ± 0.01^b^	2.16 ± 0.01^c^	< 0.001	2.25 ± 0.02	2.31 ± 0.01	2.29 ± 0.02	0.070
Vitamin B6 (mg/d)	2.59 ± 0.02^a^	2.47 ± 0.02^b^	2.24 ± 0.02^c^	< 0.001	2.55 ± 0.03^a^	2.49 ± 0.02^a^	2.27 ± 0.03^b^	< 0.001
Folic Acid (µg/d)	388.1 ± 3.2^a^	371.6 ± .3.2^b^	331.2 ± 3.2^c^	< 0.001	364.7 ± 4.6^ab^	374.0 ± 3.3^a^	352.1 ± 5.2^b^	0.001
Vitamin B12 (µg/d)	6.43 ± 0.10^a^	5.75 ± 0.10^b^	5.20 ± 0.10^c^	< 0.001	5.54 ± 0.15	5.72 ± 0.11	6.13 ± 0.17	0.104
Magnesium (mg/day)	335.2 ± 2.0^a^	329.8 ± 2.0^a^	309.9 ± 2.0^b^	< 0.001	321.7 ± 2.8	327.4 ± 2.0	325.8 ± 3.2	0.195
Calcium (mg/day)	950.0 ± 7.4^a^	946.4 ± 7.2^a^	919.2 ± 7.3^b^	0.005	892.3 ± 10.3^a^	956.6 ± 7.4^b^	966.7 ± 11.6^b^	< 0.001
Iron (mg/day)	43.7 ± 1.6	41.6 ± 1.6	39.6 ± 1.6	0.216	37.4 ± 2.3^a^	45.5 ± 1.6^b^	42.0 ± 2.6^ab^	0.004
Zinc (mg/day)	12.6 ± 0.08^a^	11.5 ± 0.08^b^	10.5 ± 0.08^c^	< 0.001	11..2 ± 0.1^a^	11.8 ± 0.1^b^	11.5 ± 0.1^ab^	< 0.001
**Food groups**^**2**^								
Whole grains (g/day)	51.8 ± 1.5^a^	69.9 ± 1.5^b^	102.2 ± 1.5^c^	< 0.001	46.2 ± 2.2^a^	76.2 ± 1.6^b^	101.4 ± 2.5^c^	< 0.001
Refined grains (g/day)	195.8 ± 3.9^a^	227.4 ± 3.8^b^	245.7 ± 3.8^c^	< 0.001	188.6 ± 5.4^a^	223.9 ± 3.9^b^	256.3 ± 3.1^c^	< 0.001
Low fat dairy products (g/day)	70.3 ± 2.6^a^	59.1 ± 2.6^b^	48.7 ± 2.6^c^	< 0.001	56.1 ± 3.7	62.7 ± 2.7	59.5 ± 4.1	0.232
High fat dairy products (g/day)	189.8 ± 3.9^a^	180.7 ± 3.8^ab^	168.3 ± 3.9^b^	0.001	178.8 ± 5.5	185.3 ± 4.0	174.7 ± 6.2	0.225
Nuts (g/day)	28.7 ± 0.6^a^	21.7 ± 0.6^b^	15.9 ± 0.6^c^	< 0.001	32.4 ± 0.9^a^	24.3 ± 0.6^b^	9.5 ± 1.0^c^	< 0.001
Legumes (g/day)	49.0 ± 1.2^a^	47.4 ± 1.2^ab^	43.6 ± 1.2^b^	0.009	38.3 ± 1.7^a^	45.8 ± 1.3^b^	56.1 ± 2.0^c^	< 0.001
Red meats (g/day)	57.5 ± 1.3^a^	54.7 ± 1.3^ab^	50.6 ± 1.3^b^	0.001	42.4 ± 1.8^a^	55.1 ± 1.3^b^	65.4 ± 2.0^c^	< 0.001
Processed meats (g/day)	76.4 ± 1.4^a^	66.3 ± 1.4^b^	60.2 ± 1.4^c^	< 0.001	59.2 ± 2.0^a^	68.9 ± 1.4^b^	74.8 ± 2.2^b^	< 0.001
Fruits (g/day)	454.4 ± 9.7^a^	477.0 ± 9.7^a^	511.2 ± 9.6^b^	< 0.001	365.5 ± 13.5^a^	479.9 ± 9.7^b^	597.1 ± 15.2^c^	< 0.001
Vegetables (g/day)	228.8 ± 4.6^a^	222.9 ± 4.5^a^	191.8 ± 4.6^b^	< 0.001	195.3 ± 6.4^a^	217.4 ± 4.7^b^	230.8 ± 7.3^b^	0.005
Caffeine (mg/day)	100.2 ± 3.5^a^	107.6 ± 3.4^ab^	117.0 ± 3.4^b^	0.003	109.3 ± 4.9	108.4 ± 3.5	107.0 ± 5.5	0.970

^1^Values are adjusted for, sex, age and total energy

^2^Values are reported as Mean ± standard Error (SE)

^3^values with different super scripts are significantly different

Table 3 presents the association between the DII and DIL and odds of developing sleep abnormalities in crude and multivariable-adjusted models in the whole population (*n* = 5925). In the fully adjusted model, subjects in the highest tertile of DIL had lower odds of sleep disorder compared with those in the lowest tertile (OR: 0.38; 95% CI: 0.17–0.85; P trend = 0.02). Also we found a significant inverse association, such that subjects in the highest tertile of DIL had lower odds of delay in falling asleep compared with those in the lowest tertile (OR = 0.66; 95% CI: 0.42–1.03, P trend = 0.05). Individuals in the highest tertile of DIL were less likely to use medications for sleep (OR: 0.60; 95% CI: 0.43–0.84, P trend = 0.002) compared with those in the lowest tertile in the crude model. However, after taking confounders into account, these associations became non-significant. In the crude or multivariable-adjusted models no other significant association was observed between DIL and sleep abnormalities (*P* > 0.05). In terms of DII, participants in the top tertile of DII had lower odds of sleep disorder compared with those in the bottom tertile in crude model (OR = 0.67; 95% CI: 0.46–0.97, P trend = 0.02). Even after adjusting for all possible confounders, this association remained significant. (OR = 0.61; 95% CI: 0.39–0.93, P trend = 0.02).

**Table 3:** The likelihood for Sleep abnormalities according to tertiles of insulin index and insulin load

Variables	Dietary insulin index			P trend	Dietary insulin load			P trend
	**T**_**1**_	**T**_**2**_	**T3**		**T**_**1**_	**T**_**2**_	**T**_**3**_	
**All participants**								
**Short sleep duration (< 5 h vs. 5–8 h)**								
** Crude**	1	0.99 (0.80–1.22)	1.08 (0.88–1.33)	0.44	1	0.96 (0.78–1.19)	1.04 (0.85–1.29)	0.66
** Model 1 **^**2**^	1	0.99 (0.79–1.23)	1.07 (0.86–1.33)	0.52	1	1.03 (0.81–1.31)	1.24 (0.83–1.84)	0.37
** Model 2 **^**3**^	1	1.06 (0.84–1.34)	1.09 (0.86–1.37)	0.46	1	0.99 (0.76–1.28)	1.21 (0.80–1.85)	0.49
** Model 3 **^**4**^	1	1.05 (0.83–1.32)	1.08 (0.85–1.35)	0.52	1	0.99 (0.76–1.28)	1.20 (0.79–1.83)	0.51
**Long sleep duration (> 8 h vs. 5–8 h)**								
** Crude**	1	1.22 (0.98–1.51)	1.16 (0.93–1.44)	0.18	1	0.98 (0.79–1.22)	1.06 (0.86–1.32)	0.55
** Model 1**	1	1.24 (0.99–1.55)	1.21 (0.97–1.52)	0.10	1	0.96 (0.75–1.23)	1.05 (0.70–1.58)	0.91
** Model 2**	1	1.26 (1.00–1.60)	1.22 (0.96–1.56)	0.10	1	0.93 (0.72–1.22)	1.04 (0.68–1.61)	0.99
** Model 3**	1	1.28 (1.01–1.63)	1.24 (0.97–1.58)	0.09	1	0.93 (0.71–1.21)	1.05 (0.68–1.63)	0.98
**Low sleep quality**								
** Crude**	1	0.75 (0.48–1.17)	0.76 (0.48–1.17)	0.21	1	0.79 (0.50–1.25)	1.01 (0.65–1.56)	0.96
** Model 1**	1	0.74 (0.45–1.17)	0.76 (0.48–1.21)	0.25	1	0.74 (0.43–1.27)	0.58 (0.24–1.39)	0.20
** Model 2**	1	0.81 (0.50–1.33)	0.71 (0.42–1.18)	0.18	1	0.57 (0.31–1.03)	0.46 (0.17–1.18)	0.07
** Model 3**	1	0.81 (0.44–1.45)	0.65 (0.35–1.21)	0.18	1	0.78 (0.39-.1.58)	0.44 (0.14–1.41)	0.20
**Insufficient sleep**								
** Crude**	1	0.97 (0.78–1.27)	1.07 (0.87–1.31)	0.52	1	0.96 (0.78–1.19)	1.04 (0.84–1.28)	0.71
** Model 1**	1	0.97 (0.78–1.20)	1.05 (0.84–1.30)	0.65	1	1.03 (0.81–1.31)	1.22 (0.83–1.81)	0.38
** Model 2**	1	1.03 (0.82–1.30)	1.06 (0.84–1.33)	0.59	1	0.99 (0.76–1.28)	1.20 (0.79–1.82)	0.51
** Model 3**	1	1.02 (0.81–1.28)	1.05 (0.83–1.32)	0.67	1	0.99 (0.76–1.28)	1.19 (0.78–1.81)	0.53
**Delay in falling asleep**								
** Crude**	1	0.82 (0.66–1.02)	0.88 (0.71–1.08)	0.23	1	0.84 (0.67–1.03)	0.94 (0.76–1.16)	0.55
** Model 1**	1	0.82 (0.66–1.01)	0.88 (0.71–1.09)	0.25	1	0.81 (0.63–1.03)	0.78 (0.52–1.16)	0.14
** Model 2**	1	0.81 (0.64–1.02)	0.85 (0.67–1.02)	0.18	1	0.75 (0.57–0.97)	0.66 (0.42–1.02)	**0.03**
** Model 3**	1	0.82 (0.65–1.05)	0.85 (0.67–1.08)	0.18	1	0.77 (0.59–1.01)	0.66 (0.42–1.03)	0.05
**Had to use medication for sleep**								
** Crude**	1	0.90 (0.65–1.26)	1.01 (0.73–1.40)	0.93	1	0.68 (0.49–0.94)	0.60 (0.43–0.84)	** < 0.01**
** Model 1**	1	0. 81 (0.57–1.14)	0.88 (0.63–1.23)	0.48	1	0.78 (0.53–1.14)	0.71 (0.36–1.37)	0.22
** Model 2**	1	0.88 (0.60–1.27)	0.86 (0.59–1.24)	0.43	1	0.69 (0.45–1.05)	0.64 (0.31–1.34)	0.13
**Sleep disorders**								
** Crude**	1	0.61 (0.41–0.89)	0.67 (0.46–0.97)	**0.02**	1	0.90 (0.61–1.32)	1.04 (0.71–1.51)	0.83
** Model 1**	1	0.61 (0.42–0.91)	0.69 (0.47–1.02)	0.052	1	0.79 (0.50–1.23)	0.60 (0.29–1.24)	0.16
** Model 2**	1	0.65 (0.43–0.98)	0.62 (0.40–0.95)	**0.02**	1	0.63 (0.38–1.02)	0.38 (0.17–0.85)	**0.01**
** Model 3**	1	0.64 (0.42–0.97)	0.61 (0.39–0.93)	**0.02**	1	0.64 (0.39–1.04)	0.38 (0.17–0.85)	**0.02**

^1^Data are odds ratio (95% CI)

^2^Adjusted for age (category), sex and total energy

^3^Adjusted for age (category), sex, total energy, BMI (category), marital status (category), physical activity(continuous), smoking status (category), education status (category), job status (category), anxiety (continuous), stress (continuous), caffeine (continuous), duration of Cell phone use (continuous) and Watching television & movie (continuous)

^4^further controlled for sleep medication use

The interaction term between DII and gender was not significant for any outcomes but in terms of DIL and gender, the interaction was significant just for “delay in falling asleep” P = 0.02 not other outcomes. The association between DII and DIL and sleep abnormalities, based on sex, is indicated in Tables 4 and 5. Men in the highest tertile of DIL had lower odds of sleep medication use compared with those in the lowest tertile, in crude model (OR = 0.60; 95% CI: 0.36–1.00, P trend = 0.04). Even after adjusting for all possible confounders, this association remained significant. (OR = 0.28; 95% CI: 0.08–0.91, P trend = 0.01) (Table 4).

**Table 4:** The likelihood for Sleep abnormalities according to tertiles of insulin index and insulin load in male group

Variables	Dietary insulin index			P trend	Dietary insulin load			P trend
	**T**_**1**_	**T**_**2**_	**T3**		**T**_**1**_	**T**_**2**_	**T**_**3**_	
**Short sleep duration (< 5 h vs. 5–8 h)**								
** Crude**	1	1.11 (0.84–1.47)	1.21 (0.92–1.59)	0.16	1	1.08 (0.82–1.44)	1.10 (0.83–1.45)	0.52
** Model 1 **^**2**^	1	1.11 (0.84–1.48)	1.22 (0.92–1.61)	0.17	1	1.17 (0.85–1.60)	1.34 (0.80–2.24)	0.24
** Model 2 **^**3**^	1	1.24 (0.91–1.68)	1.25 (0.92–1.69)	0.15	1	1.09 (0.78–1.52)	1.31 (0.76–2.26)	0.36
** Model 3 **^**4**^	1	1.23 (0.90–1.67)	1.23 (0.91–1.67)	0.17	1	1.07 (0.76–1.50)	1.26 (0.73–1.18)	0.44
**Long sleep duration (> 8 h vs. 5–8 h)**								
** Crude**	1	1.19 (0.83–1.69)	0.98 (0.68–1.42)	0.93	1	0.97 (0.67–1.42)	1.25 (0.87–1.78)	0.20
** Model 1**	1	1.25 (0.87–1.79)	1.07 (0.73–1.55)	0.72	1	0.87 (0.57–1.32)	0.90 (0.46–1.74)	0.67
** Model 2**	1	1.34 (0.91–2.00)	1.17 (0.78–1.76)	0.43	1	1.04 (0.66–1.65)	1.07 (0.52–2.19)	0.84
** Model 3**	1	1.34 (0.90–2.00)	1.17 (0.78–1.76)	0.44	1	1.06 (0.67–1.68)	1.09 (0.53–2.55)	0.79
**Low sleep quality**								
** Crude**	1	0.73 (0.32–1.65)	0.91 (0.43–1.96)	0.81	1	0.81 (0.33–2.01)	1.56 (0.72–3.41)	0.21
** Model 1**	1	0.83 (0.36–1.89)	1.06 (0.48–2.33)	0.88	1	0.52 (0.19–1.40)	0.31 (0.06–1.44)	0.12
** Model 2**	1	1.08 (0.43–2.75)	1.13 (0.45–2.81)	0.78	1	0.31 (0.10–1.01)	0.22 (0.04–1.16)	0.06
** Model 3**	1	1.15 (0.34–3.90)	1.51 (0.47–4.88)	0.49	1	0.84 (0.22–3.27)	0.67 (0.08–5.63)	0.71
**Insufficient sleep**								
** Crude**	1	1.10 (0.83–1.45)	1.21 (0.92–1.59)	0.15	1	1.09 (0.82–1.44)	1.08 (0.82–1.42)	0.60
** Model 1**	1	1.09 (0.82–1.45)	1.21 (0.91–1.60)	0.18	1	1.18 (0.86–1.62)	1.35 (0.81–2.25)	0.22
** Model 2**	1	1.21 (0.90–1.64)	1.24 (0.92–1.67)	0.16	1	1.08 (0.77–1.52)	1.30 (0.76–2.24)	0.36
** Model 3**	1	1.20 (0.89–1.63)	1.22 (0.91–1.66)	0.19	1	1.07 (0.76–1.50)	1.25 (0.73–2.16)	0.44
**Delay in falling asleep**								
** Crude**	1	0.97 (0.69–1.36)	0.97 (0.69–1.35)	0.85	1	0.93 (0.65–1.33)	1.34 (0.96–1.86)	0.06
** Model 1**	1	1.01 (0.72–1.41)	1.02 (0.72–1.43)	0.90	1	0.94 (0.63–1.40)	1.34 (0.73–2.46)	0.47
** Model 2**	1	1.04 (0.72–1.49)	0.95 (0.66–1.37)	0.81	1	0.83 (0.55–1.27)	1.14 (0.60–2.18)	0.88
** Model 3**	1	1.08 (0.75–1.56)	1.00 (0.68–1.43)	0.95	1	0.91 (0.59–1.40)	1.23 (0.67–2.50)	0.59
**Had to use medication for sleep**								
** Crude**	1	0.88 (0.51–1.52)	1.12 (0.67–1.86)	0.65	1	0.58 (0.34–0.98)	0.60 (0.36–1.00)	**0.04**
** Model 1**	1	0.84 (0.48–1.45)	1.00 (0.59–1.70)	0.95	1	0.50 (0.27–0.91)	0.33 (0.11–0.95)	**0.02**
** Model 2**	1	0.85 (0.46–1.58)	0.94 (0.52–1.69)	0.85	1	0.43 (0.22–0.85)	0.28 (0.08–0.91)	**0.01**
**Sleep disorders**								
** Crude**	1	0.35 (0.16–0.75)	0.64 (0.34–1.18)	0.12	1	0.66 (0.30–1.45)	1.52 (0.80–2.88)	0.14
** Model 1**	1	0.38 (0.18–0.83)	0.74 (0.39–1.40)	0.01	1	0.47 (0.20–1.10)	0.51 (0.15–1.72)	0.20
** Model 2**	1	0.35 (0.14–0.84)	0.77 (0.39–1.54)	0.36	1	0.30 (0.11–0.77)	0.39 (0.11–1.41)	0.09
** Model 3**	1	0.33 (0.1–0.81)	0.77 (0.38–1.56)	0.35	1	0.32 (0.12–0.84)	0.45 (0.12–1.67)	0.14

^1^Data are odds ratio (95% CI)

^2^Adjusted for age (category), sex and total energy

^3^Adjusted for age (category), sex, total energy, BMI (category), marital status (category), physical activity(continuous), smoking status (category), education status (category), job status (category), anxiety (continuous), stress (continuous), caffeine (continuous), duration of Cell phone use (continuous) and Watching television & movie (continuous)

^4^further controlled for sleep medication use

**Table 5:** The likelihood for Sleep abnormalities according to tertiles of insulin index and insulin load in female group

Variables	Dietary insulin index			P trend	Dietary insulin load			P trend
	**T**_**1**_	**T**_**2**_	**T3**		**T**_**1**_	**T**_**2**_	**T**_**3**_	
**Short sleep duration (< 5 h vs. 5–8 h)**								
** Crude**	1	0.85 (0.62–1.18)	0.91 (0.66–1.26)	0.57	1	0.79 (0.57–1.01)	0.95 (0.67–1.31)	0.70
** Model 1 **^**2**^	1	0.84 (0.60–1.17)	0.90 (0.64–1.26)	0.54	1	0.86 (0.59–1.27)	1.11 (0.60–2.07)	0.98
** Model 2 **^**3**^	1	0.87 (0.61–1.24)	0.92 (0.64–1.32)	0.65	1	0.86 (0.58–1.30)	1.10 (0.57–2.12)	0.99
** Model 3 **^**4**^	1	0.87 (0.61–1.23)	0.91 (0.63–1.30)	0.59	1	0.86 (0.57–1.30)	1.10 (0.57–2.13)	0.98
**Long sleep duration (> 8 h vs. 5–8 h)**								
** Crude**	1	1.21 (0.92–1.59)	1.28 (0.97–1.69)	0.07	1	1.03 (0.79–1.34)	1.04 (0.79–1.36)	0.76
** Model 1**	1	1.23 (0.93–1.63)	1.29 (0.97–1.71)	0.08	1	1.03 (0.76–1.40)	1.16 (0.70–1.95)	0.61
** Model 2**	1	1.25 (0.92–1.69)	1.26 (0.93–1.71)	0.14	1	0.89 (0.64–1.23)	1.08 (0.62–1.86)	0.96
** Model 3**	1	1.25 (0.92–1.69)	1.25 (0.92–1.70)	0.15	1	0.88 (0.63–1.22)	1.08 (0.62–1.87)	0.96
**Low sleep quality**								
** Crude**	1	0.75 (0.44–1.27)	0.69 (0.40–1.20)	0.18	1	0.82 (0.48–1.41)	0.87 (0.50–1.49)	0.59
** Model 1**	1	0.68 (0.39–1.18)	0.64 (0.36–1.14)	0.13	1	0.96 (0.50–1.82)	0.87 (0.30–2.55)	0.82
** Model 2**	1	0.75 (0.41–1.37)	0.60 (0.32–1.13)	0.11	1	0.84 (0.42–1.72)	0.85 (0.26–2.76)	0.72
** Model 3**	1	0.69 (0.32–1.45)	0.50 (0.23–1.09)	0.08	1	1.05 (0.44–2.49)	0.57 (0.13–2.44)	0.58
**Insufficient sleep**								
** Crude**	1	0.83 (0.60–1.15)	0.88 (0.64–1.22)	0.44	1	0.79 (0.57–1.09)	0.94 (0.68–1.29)	0.67
** Model 1**	1	0.81 (0.58–1.13)	0.86 (0.62–1.20)	0.39	1	0.85 (0.58–1.25)	1.07 (0.57–1.98)	0.92
** Model 2**	1	0.84 (0.59–1.19)	0.87 (0.61–1.25)	0.46	1	0.87 (0.58–1.30)	1.08 (0.56–2.08)	0.98
** Model 3**	1	0.84 (0.59–1.19)	0.86 (0.60–1.23)	0.42	1	0.87 (0.58–1.30)	1.08 (0.56–2.09)	0.99
**Delay in falling asleep**								
** Crude**	1	0.73 (0.55–0.96)	0.83 (0.64–1.09)	0.18	1	0.82 (0.63–1.08)	0.77 (0.59–1.02)	0.06
** Model 1**	1	0.70 (0.53–0.93)	0.80 (0.60–1.06)	0.12	1	0.74 (0.54–1.02)	0.51 (0.29–0.88)	**0.01**
** Model 2**	1	0.67 (0.49–0.92)	0.79 (0.58–1.08)	0.13	1	0.69 (0.49–0.98)	0.43 (0.24–0.79)	** < 0.01**
** Model 3**	1	0.68 (0.50–0.93)	0.78 (0.57–1.06)	0.11	1	0.69 (0.49–0.98)	0.41 (0.22–0.75)	** < 0.01**
**Had to use medication for sleep**								
** Crude**	1	0.90 (0.59–1.37)	0.95 (0.62–1.45)	0.82	1	0.78 (0.52–1.16)	0.63 (0.41–0.97)	**0.03**
** Model 1**	1	0.78 (0.50–1.21)	0.80 (0.52–1.25)	0.35	1	1.08 (0.66–1.77)	1.22 (0.52–2.87)	0.65
** Model 2**	1	0.91 (0.57–1.47)	0.83 (0.51–1.36)	0.47	1	0.99 (0.57–1.70)	1.25 (0.49–3.22)	0.74
**Sleep disorders**								
** Crude**	1	0.74 (0.47–1.16)	0.69 (0.43–1.10)	0.11	1	1.05 (0.67–1.65)	0.89 (0.55–1.43)	0.66
** Model 1**	1	0.72 (0.45–1.15)	0.67 (0.41–1.09)	0.75	1	1.02 (0.60–1.72)	0.67 (0.27–1.68)	0.54
** Model 2**	1	0.82 (0.50–1.35)	0.56 (0.32–0.98)	**0.04**	1	0.88 (0.49–1.59)	0.37 (0.13–1.07)	0.13
** Model 3**	1	0.81 (0.49–1.34)	0.56 (0.32–0.97)	**0.04**	1	0.89 (0.49–1.61)	0.36 (0.12–1.04)	0.12

^1^Data are odds ratio (95% CI)

^2^Adjusted for age (category), sex and total energy

^3^Adjusted for age (category), sex, total energy, BMI (category), marital status (category), physical activity (continuous), smoking status (category), education status (category), job status (category), anxiety (continuous), stress, (continuous) caffeine (continuous), duration of Cell phone use (continuous) and Watching television & movie (continuous)

^4^further controlled for sleep medication use

After adjusting for all confounders, women in the top tertile of DIL had lower odds of delay in falling asleep compared with those in the first tertile (OR = 0.41; 95% CI: 0.22–0.75; P trend =  < 0.01) (Table 5). Although a significant inverse association was seen between DIL and odds of sleep medication use in crude model among women (OR = 0.63; 95% CI: 0.41–0.97; P trend = 0.03), the association was non-significant after controlling for all potential confounders (OR = 1.25; 95% CI: 0.49–3.22; P trend = 0.74). In terms of DII, women in the highest tertile had a lower odds of sleep disorder compared with those in the lowest tertile in the fully adjusted model (OR: 0.56; 95% CI: 0.32–0.97; P trend = 0.04).

The interaction term between DII and BMI was significant for “sleep quality” *P* = 0.007 and “Had to use medication for sleep “ *P* = 0.04 also in terms of DIL and gender, the interaction was significant for “sleep quality” *P* = 0.009 and “Had to use medication for sleep “ *P* = 0.05 too. The interaction term between DII/DIL and BMI for other outcomes wasn’t significant. Findings from stratified analysis based on BMI status are provided in Tables 6 and 7. In crude or multivariable-adjusted models, there was no significant association with sleep abnormalities among normal-weight persons (BMI 25 kg/m2) (*P* > 0.05). However, among overweight or obese people (participants with BMI ≥ 25 kg/m^2^), we found an inverse significant association between DIL and odds of sleep disorder (OR = 0.28; 95% CI: 0.10, 0.78, P trend = 0.01), and delay in falling asleep (OR = 0.50; 95% CI: 0.28, 0.89, P trend = 0.02). We also observed a significant association between DIL and odds of medication use in the crude model (OR = 0.47; 95% CI: 0.31, 0.73, P-trend ≤ 0.001). However, this association disappeared when potential confounders were adjusted. In terms of DII, greater DII was significantly associated with lower odds of low sleep quality (OR = 0.35; 95% CI: 0.16, 0.78, P trend = 0.01), sleep disorder (OR = 0.43; 95% CI: 0.24, 0.75, P trend = 0.002); and sleep medication use (OR = 0.62; 95% CI: 0.39, 0.99, P trend = 0.05) after adjustment for all possible confounders. Also, individuals with the highest DII had a higher chance for having long-sleep duration compared to those in the lowest tertile (OR = 1.43; 95% CI: 1.04, 1.97, P trend = 0.03).

**Table 6:** The likelihood for Sleep abnormalities according to tetiles of insulin index and insulin load in BMI < 25

Variables	Dietary insulin index			P trend	Dietary insulin load			P trend
	**T**_**1**_	**T**_**2**_	**T3**		**T**_**1**_	**T**_**2**_	**T**_**3**_	
**Short sleep duration (< 5 h vs. 5–8 h)**								
** Crude**	1	0.93 (0.64–1.35)	1.03 (0.72–1.47)	0.85	1	0.76 (0.53–1.09)	0.92 (0.64–1.31)	0.63
** Model 1 **^**2**^	1	0.92 (0.63–1.34)	1.01 (0.70–1.45)	0.95	1	0.88 (0.58–1.34)	1.37 (0.69–2.74)	0.64
** Model 2 **^**3**^	1	0.95 (0.64–1.42)	1.02 (0.69–1.50)	0.92	1	0.87 (0.56–1.35)	1.35 (0.65–2.79)	0.70
** Model 3 **^**4**^	1	0.95 (0.64–1.41)	0.98 (0.66–1.46)	0.94	1	0.89 (0.57–1.39)	1.39(0.66–2.88)	0.63
**Long sleep duration (> 8 h vs. 5–8 h)**								
** Crude**	1	1.11 (0.80–1.55)	0.94 (0.67–1.32)	0.73	1	1.01 (0.72–1.42)	1.18 (0.84–1.65)	0.33
** Model 1**	1	1.11 (0.79–1.56)	0.98 (0.69–1.39)	0.93	1	0.86 (0.58–1.26)	0.85 (0.45–1.62)	0.54
** Model 2**	1	1.24 (0.87–1.79)	1.00 (0.68–1.46)	0.99	1	0.83 (0.55–1.26)	0.99 (0.50–1.96)	0.78
** Model 3**	1	1.24 (0.87–1.79)	1.00 (0.68–1.45)	0.99	1	0.83 (0.55–1.26)	1.00 (0.51–1.99)	0.80
**Low sleep quality**								
** Crude**	1	1.49 (0.66–3.38)	1.67 (0.75–3.70)	0.21	1	0.98 (0.41–2.34)	1.90 (0.88–4.13)	0.08
** Model 1**	1	1.76 (0.47–4.18)	2.16 (0.93–5.02)	0.07	1	1.04 (0.40–2.72)	1.22 (0.29–5.09)	0.80
** Model 2**	1	1.82 (0.73–4.53)	2.05 (0.83–5.04)	0.12	1	0.84 (0.29–2.42)	0.91 (0.19–4.32)	0.87
** Model 3**	1	1.61 (0.47–5.51)	1.92 (0.57–6.45)	0.29	1	2.01 (0.48–8.32)	1.48 (0.18–12.15)	0.61
**Insufficient sleep**								
** Crude**	1	0.92 (0.64–1.33)	1.04 (0.73–1.48)	0.82	1	0.76 (0.53–1.09)	0.90 (0.63–1.29)	0.56
** Model 1**	1	0.91 (0.63–1.32)	1.00 (0.70–1.44)	0.97	1	0.89 (0.59–1.35)	1.38 (0.69–2.73)	0.61
** Model 2**	1	0.92 (0.62–1.36)	1.01 (0.69–1.49)	0.94	1	0.88 (0.56–1.36)	1.31 (0.63–2.67)	0.72
** Model 3**	1	0.91 (0.61–1.35)	0.98 (0.66–1.44)	0.92	1	0.90 (0.58–1.40)	1.34 (0.65–2.77)	0.65
**Delay in falling asleep**								
** Crude**	1	0.93 (0.66–1.32)	1.00 (0.71–1.41)	0.99	1	0.84 (0.59–1.21)	1.22 (0.86–1.71)	0.23
** Model 1**	1	0.97 (0.67–1.38)	1.06 (0.75–1.51)	0.73	1	0.85 (0.57–1.27)	1.13 (0.59–2.15)	0.94
** Model 2**	1	0.98 (0.67–1.44)	1.05 (0.72–1.54)	0.78	1	0.79 (0.51–1.22)	1.09 (0.54–2.18)	0.93
** Model 3**	1	0.98 (0.67–1.44)	1.02 (0.69–1.50)	0.92	1	0.81 (0.52–1.26)	1.05 (0.52–2.12)	0.88
**Had to use medication for sleep**								
** Crude**	1	1.35 (0.77–2.39)	1.31 (0.74–2.31)	0.35	1	0.74 (0.42–1.28)	0.85 (0.49–1.47)	0.56
** Model 1**	1	1.37 (0.76–2.47)	1.36 (0.75–2.46)	0.32	1	0.83 (0.44–1.58)	0.75 (0.25–2.24)	0.56
** Model 2**	1	1.53 (0.78–2.99)	1.55 (0.80–3.00)	0.20	1	0.64 (0.31–1.33)	0.74 (0.22–2.49)	0.43
**Sleep disorders**								
** Crude**	1	0.57 (0.28–1.16)	0.94 (0.51–1.74)	0.82	1	1.02 (0.50–2.06)	1.47 (0.75–2.85)	0.24
** Model 1**	1	0.62 (0.30–1.28)	0.56 (0.35–0.91)	0.85	1	0.95 (0.43–2.08)	0.93 (0.27–3.21)	0.90
** Model 2**	1	0.71 (0.33–1.53)	1.20 (0.59–2.43)	0.63	1	0.94 (0.40–2.22)	0.68 (0.17–2.70)	0.62
** Model 3**	1	0.66 (0.30–1.43)	1.08 (0.53–2.21)	0.83	1	1.01 (0.42–2.39)	0.69 (0.17–2.75)	0.66

^1^Data are odds ratio (95% CI)

^2^Adjusted for age (category), sex and total energy

^3^Adjusted for age (category), sex, total energy, BMI (category), marital status (category), physical activity (continuous), smoking status (category), education status (category), job status (category), anxiety (continuous), stress, (continuous) caffeine (continuous), duration of Cell phone use (continuous) and Watching television & movie (continuous)

^4^further controlled for sleep medication use

**Table 7:** The likelihood for Sleep abnormalities according to tertiles of insulin index and insulin load in BMI ≥ 25

Variables	Dietary insulin index			P trend	Dietary insulin load			P trend
	**T**_**1**_	**T**_**2**_	**T3**		**T**_**1**_	**T**_**2**_	**T**_**3**_	
**Short sleep duration (< 5 h vs. 5–8 h)**								
** Crude**	1	1.00 (0.77–1.29)	1.07 (0.82–1.38)	0.61	1	1.07 (0.82–1.40)	1.10 (0.85–1.43)	0.45
** Model 1 **^**2**^	1	1.00 (0.77–1.31)	1.06 (0.81–1.38)	0.66	1	1.07 (0.79–1.45)	1.13 (0.69–1.84)	0.60
** Model 2 **^**3**^	1	1.11 (0.84–1.48)	1.14 (0.86–1.51)	0.37	1	1.06 (0.77–1.46)	1.16 (0.69–1.94)	0.57
** Model 3 **^**4**^	1	1.09 (0.82–1.45)	1.12 (0.84–1.50)	0.41	1	1.05 (0.76–1.44)	1.12 (0.67–1.88)	0.66
**Long sleep duration (> 8 h vs. 5–8 h)**								
** Crude**	1	1.29 (0.96–1.73)	1.34 (1.00–1.80)	0.05	1	0.94 (0.71–1.25)	0.97 (0.73–1.28)	0.83
** Model 1**	1	1.32 (0.98–1.78)	1.38 (1.02–1.87)	**0.03**	1	1.01 (0.73–1.40)	1.16 (0.68–1.98)	0.65
** Model 2**	1	1.30 (0.94–1.80)	1.43 (1.04–1.97)	**0.03**	1	1.01 (0.71–1.43)	1.09 (0.62–1.93)	0.80
** Model 3**	1	1.30 (0.94–1.80)	1.43 (1.04–1.97)	**0.03**	1	1.01 (0.71–1.42)	1.08 (0.61–1.92)	0.81
**Low sleep quality**								
** Crude**	1	0.53 (0.30–0.92)	0.43 (0.23–0.78)	** < 0.01**	1	0.73 (0.41–1.28)	0.68 (0.39–1.20)	0.18
** Model 1**	1	0.48 (0.27–0.85)	0.40 (0.21–0.72)	** < 0.01**	1	0.59 (0.30–1.15)	0.30 (0.09–0.96)	0.04
** Model 2**	1	0.55 (0.30–1.02)	0.38 (0.20–0.75)	** < 0.01**	1	0.52 (0.24–1.09)	0.30 (0.08–1.07)	0.05
** Model 3**	1	0.55 (0.26–1.16)	0.35 (0.16–0.78)	**0.01**	1	0.64 (0.27–1.52)	0.24 (0.05–1.13)	0.09
**Insufficient sleep**								
** Crude**	1	0.97 (0.75–1.26)	1.04 (0.80–1.34)	0.77	1	1.08 (0.83–1.40)	1.11 (0.85–1.43)	0.43
** Model 1**	1	0.98 (0.75–1.28)	1.03 (0.79–1.34)	0.81	1	1.07 (0.79–1.44)	1.11 (0.68–1.81)	0.63
** Model 2**	1	1.08 (0.82–1.44)	1.10 (0.83–1.46)	0.51	1	1.06 (0.77–1.46)	1.16 (0.69–1.93)	0.57
** Model 3**	1	1.07 (0.80–1.42)	1.08 (0.82–1.44)	0.56	1	1.05 (0.76–1.44)	1.12 (0.67–1.87)	0.67
**Delay in falling asleep**								
** Crude**	1	0.74 (0.57–0.97)	0.78 (0.60–1.02)	0.07	1	0.81 (0.62–1.06)	0.82 (0.63–1.07)	0.14
** Model 1**	1	0.72 (0.54–0.95)	0.76 (0.57–1.00)	0.05	1	0.75 (0.55–1.04)	0.60 (0.35–1.01)	**0.04**
** Model 2**	1	0.72 (0.53–0.97)	0.74 (0.55–1.00)	0.05	1	0.72 (0.51–1.01)	0.48 (0.27–0.85)	**0.01**
** Model 3**	1	0.74 (0.57–1.00)	0.76 (0.56–1.02)	0.07	1	0.75 (0.53–1.05)	0.50 (0.28–0.89)	**0.02**
**Had to use medication for sleep**								
** Crude**	1	0.73 (0.48–1.10)	0.81 (0.54–1.22)	0.31	1	0.66 (0.44–0.98)	0.47 (0.31–0.73)	** < 0.01**
** Model 1**	1	0.61 (0.40–0.94)	0.66 (0.43–1.00)	**0.05**	1	0.76 (0.47–1.23)	0.63 (0.27–1.48)	0.24
** Model 2**	1	0.68 (0.43–1.08)	0.62 (0.39–0.99)	**0.05**	1	0.78 (0.46–1.31)	0.65 (0.25–1.68)	0.32
**Sleep disorders**								
** Crude**	1	0.56 (0.35–0.90)	0.49 (0.30–0.80)	** < 0.01**	1	0.82 (0.51–1.34)	0.91 (0.57–1.44)	0.68
** Model 1**	1	0.56 (0.35–0.91)	0.45 (0.30–0.82)	** < 0.01**	1	0.68 (0.38–1.17)	0.43 (0.17–1.09)	0.07
** Model 2**	1	0.62 (0.37–1.03)	0.43 (0.24–0.75)	** < 0.01**	1	0.53 (0.29–0.97)	0.29 (0.10–0.79)	**0.01**
** Model 3**	1	0.62 (0.37–1.03)	0.43 (0.24–0.75)	** < 0.01**	1	0.53 (0.29–0.97)	0.28 (0.10–0.78)	**0.01**

^1^Data are odds ratio (95% CI)

^2^Adjusted for age (category), sex and total energy

^3^Adjusted for age (category), sex, total energy, BMI (category), marital status (category), physical activity (continuous), smoking status (category), education status (category), job status (category), anxiety (continuous), stress (continuous) caffeine (continuous), duration of Cell phone use (continuous) and Watching television & movie (continuous)

^4^further controlled for sleep medication use

## Discussion

As far as we know, the current study is the first investigation on the association between dietary insulin index/load and sleep quality and quantity. Our findings revealed that participants with greater DIL were less likely to have sleep disorder. In terms of DII, participants in the top tertile of DII had lower odds of sleep disorder compared with those in the bottom tertile. However, men in the top tertile of DIL had lower odds of sleep medication use compared with those in the bottom tertile. In addition, women with greater dietary insulin load diet were less likely to have delay in falling asleep. Overweight or obese participants (BMI ≥ 25 kg/m^2^) with the highest DII had greater odds for having long-sleep duration, lower odds of sleep medication use and low sleep quality than those with the lowest DII. In terms of DIL, overweight or obese participants (BMI ≥ 25 kg/m^2^) with greater DIL were less likely to have delay in falling asleep.

Insufficient sleep duration and/or quality is a global public health problem [1]. Several factors including dietary intakes [13] can affect the quality and quantity of sleep. Dietary carbohydrates construct a major part of human diet. Although previous research has investigated at the relationship between dietary GI and GL and sleep quality/quantity [17, 21], no research has looked at the relationship between DIL and DII and sleep disturbances. Data on the relationship between dietary carbohydrate consumption and sleep duration, on the other hand, is conflicting. While some studies have demonstrated unfavorable effect of higher carbohydrate intake on sleep duration [38, 39], others showed a positive relationship between carbohydrate consumption and sleep length [40, 41]. We observed that participants with greater dietary insulin load were less likely to have delay in falling asleep in the current study. In line with our findings, Afaghi et al. observed a significant reduction in mean sleep onset latency with a high-GI diet consumed 4 h before bedtime, compared with a low-GI meal, on 12 healthy young men [17]. There was no evidence of a link between DII and DIL and sleep duration (short or long sleep duration). This was in agreement with the study of Daniel et al., who did a cross-sectional study on nine basketball male athletes and reported no significant effect of low GI diet on sleep duration [42]. In contrast, Yoneyama et al. reported an inverse association between a high dietary GI and risk of poor sleep [20]. Gangwisch et al. reported that high-GI diets might be a risk factor for insomnia [43]. In a cross-sectional study with a large sample of Iranian individuals, Mohammadi et al. found a significant relationship between dietary GL, but not GI, and sleep duration [21]. Other studies have found a similar favorable connection with dietary GL and sleep duration [19]. As previously stated, the focus of all published investigations has been on dietary GI and GL rather than DIL and DII and sleep quality/quantity. Dietary GI and GL do not account for other components that influence insulin secretion, whereas DII and DIL measure postprandial insulin responses to all dietary elements, including amino acids and lipids.

As a hypothesis, insulin raises the ratio of plasma tryptophan to other large neutral amino acids (LNAAs) like leucine, isoleucine, phenylalanine, valine, and tyrosine by stimulating the absorption of LNAAs into skeletal muscle, with the exception of tryptophan [44]. Furthermore, plasma glucose concentrations and the ratio of tryptophan to other LNAAs have a positive dose–response relationship [45]. There is a competitive transfer of these amino acids through the blood–brain barrier, increasing the ratio of tryptophan to other LNAAs leads to increased tryptophan transfer and consequently the synthesis of 5-hydroxytryptamine in the brain [44]. It is suggested that additional tissue-specific enzymes can convert this 5-hydroxytryptamine to serotonin and melatonin. Eventually, a rise in melatonin production is related with a decrease in delay in falling asleep [46].

In the present study, we found a sex and BMI dependent associations between DII and DIL and sleep quality and quantity. The fundamental mechanism for this gender disparity is unknown; however, the role of sex hormones in sleep quality could explain it in part. Another factor for the sex difference could be because women's stated dietary intakes are more accurate than men's [47]. One of the possible reasons for the difference in findings by BMI status might be the relationship between obesity and sleep quality and quantity. Obese people's sleep disorders, such as obstructive sleep apnea and obesity hypoventilation syndrome, would inevitably influence the quantity and quality of their sleep [48]. Moreover, people with obesity are more likely to report insomnia or trouble sleeping than normal weight individuals [49].

The current study has a number of advantages. As far as we know this is the first study examining the association between dietary insulinemic potential and sleep quality and quantity. Additionally, we examined these associations in a large sample of adults and we considered several possible confounders in our investigation. BMI and sex-stratified analysis was done. In addition, strengths of this study are the use of a validated FFQ for dietary assessment. However, this investigation had several limitations. The main limitation is that the study design was cross-sectional, which prevents us from inferring causality. Therefore, further analysis on the prospective data of YaHS-TAMYZ or other studies are required to confirm our findings. The dietary intakes, sleep quality, and sleep duration were all measured using self-reported data, which are susceptible to measurement errors. However, such measurement errors would attenuate the risk estimates; as a result, the associations observed in this study could be substantially stronger. The DII and DIL were computed using internationally available data. This should be considered while interpreting our findings.

In conclusion, we found evidence indicating that participants with greater dietary insulin load and dietary insulin index were less likely to have sleep disorder. We found no evidence of a link between dietary insulinemic potential and sleep quantity. More research, particularly with a prospective design, in other populations is needed to confirm these findings.

## Supplementary Information

**Additional file 1:** **Supplementary Table 1.** Food insulin index (FII) of all 178 FFQ food items according to theprevious studies.

## Data Availability

The data that support the findings of this study are available from the corresponding author, [author initials], upon reasonable request.

## Abbreviations

DII: Dietary Insulin Index
DIL: Dietary Insulin Load
BMI: Body Mass Index
