# Supplementary material for: Dietary insulinemic potential, sleep quality and quantity in Iranian adults: Yazd health study and TAMYZ study
Source: BMC Nutr. 2023 Jul 25;9:92. doi: 10.1186/s40795-023-00745-6 (PMC10369837; doi:10.1186/s40795-023-00745-6)
Supplement: Supplementary file 1 — Additional file 1: Supplementary Table 1. Food insulin index (FII) of all 178 FFQ food items according to theprevious studies. [file 40795_2023_745_MOESM1_ESM.docx]

Supplementary Table 1- Food insulin index (FII) of all 178 FFQ food items according to the previous studies.

| Row | Food item | The source of FII value from previous studies^a^ | FII per 1,000 kJ or 239 kcal^b^ |
| --- | --- | --- | --- |
| 1 | White bread (Lavash) | White bread^c^ | 73 ± 5 |
| 2 | White bread (Barbari) | White bread^c^ | 73 ± 5 |
| 3 | White bread (Sangak) | White bread^c^ | 73 ± 5 |
| 4 | White bread (Taftoon) | White bread^c^ | 73 ± 5 |
| 5 | Grain bread (baguette) | Grain bread^c^ | 41 ± 4 |
| 6 | Grain bread (toast) | Grain bread^c^ | 41 ± 4 |
| 7 | White rice (cooked) | White rice (cooked)^c^ | 58 ± 9 |
| 8 | Spiral pasta (cooked) | Spiral pasta (cooked)^c^ | 29 ± 4 |
| 9 | Boiled potato | Boiled potato^c^ | 88 ± 8 |
| 10 | French fries | French fries^c^ | 57 ± 6 |
| 11 | White bread (Khoshk) | White bread^c^ | 73 ± 5 |
| 12 | White bread (Korno) | White bread^c^ | 73 ± 5 |
| 13 | Vermishel | Spiral pasta (cooked)^c^ | 29 ± 4 |
| 14 | Reshte | Spiral pasta (cooked)^c^ | 29 ± 4 |
| 15 | Biscuit (Saghe talayi) | Fat-free blueberry muffin^c^ | 69 ±6 |
| 16 | Biscuit | Fat-free blueberry muffin^c^ | 69 ±6 |
| 17 | Cake Yazdi | Fat-free blueberry muffin^c^ | 69 ±6 |
| 18 | Cake | Fat-free blueberry muffin^c^ | 69 ±6 |
| 19 | Homemade Cake | Fat-free blueberry muffin^c^ | 69 ±6 |
| 20 | Corn | All-Bran Complete Wheat Flakes^c^ | 55 ± 7 |
| 21 | Barley | All-Bran Complete Wheat Flakes^c^ | 55 ± 7 |
| 22 | Bulgur | All-Bran Complete Wheat Flakes^c^ | 55 ± 7 |
| 23 | Lentil | Lentils^d^ | 42 ± 9 |
| 24 | Beans (cooked) | Baked beans^c^ | 88 ± 14 |
| 25 | Peas | Peas, steamed^e^ | 37 ± 8 |
| 26 | Fava beans | Baked beans^c^ | 88 ± 14 |
| 27 | Soy | Tofu^c^ | 21 ± 4 |
| 28 | Cotyledon | Peas, steamed^e^ | 37 ± 8 |
| 29 | Organ meat (cow) | Beef^c^ | 37 ± 12 |
| 30 | Organ meat (sheep) | Beef^c^ | 37 ± 12 |
| 31 | Mince meat | Beef^c^ | 37 ± 12 |
| 32 | Poultry | Chicken, panfried with skin^c^ | 19 ± 4 |
| 33 | Poultry (with skin) | Chicken, panfried with skin^c^ | 19 ± 4 |
| 34 | Fish | Fish^c^ | 43 ± 13 |
| 35 | Fish (tuna) | Fish^c^ | 43 ± 13 |
| 36 | Hamburgers | Ham, shaved (Coles)^e^ | 19 ± 11 |
| 37 | Sausage | Bologna^c^ | 11 ± 2 |
| 38 | Kalbas | Bologna^c^ | 11 ± 2 |
| 39 | Egg | Egg^c^ | 23 ± 4 |
| 40 | Liver (sheep, cow) | Beef^c^ | 37 ± 12 |
| 41 | Stomach (sheep, cow) | Beef^c^ | 37 ± 12 |
| 42 | Tongue (sheep, cow) | Lamb^e^ | 41 ± 19 |
| 43 | Brain (sheep, cow) | Lamb^e^ | 41 ± 19 |
| 44 | Head (sheep, cow) | Lamb^e^ | 41 ± 19 |
| 45 | Leg (sheep, cow) | Lamb^e^ | 41 ± 19 |
| 46 | Pizza | Pizza^c^ | 47 ± 4 |
| 47 | Low fat milk | 1%-Fat milk^c^ | 34 ± 4 |
| 48 | High fat milk | Milk^c^ | 24 ± 3 |
| 49 | Chocolate milk | Chocolate milk^e^ | 46 ± 23 |
| 50 | Yogurt | Yoghurt^e^ | 46 ± 19 |
| 51 | Cheese | Cottage cheese^c^ | 40 ± 7 |
| 52 | Cream cheese | Cream cheese^c^ | 18 ± 6 |
| 53 | Yogurt drink (dough) | Yogurt, strawberry low-fat^c^ | 84 ± 9 |
| 54 | Cream | Cream^e^ | 8 ± 8 |
| 55 | Traditional ice cream | Ice cream^d^ | 65 ± 9 |
| 56 | Ice cream | Ice cream^d^ | 65 ± 9 |
| 57 | Butter | Butter^c^ | 2 ± 1 |
| 58 | Kashk (curd) | Yoghurt^e^ | 46 ± 19 |
| 59 | Lettuce | Cauliflower, steamede^e^ | 48 ± 9 |
| 60 | Tomato | Broccoli^e^ | 29 ± 8 |
| 61 | Cucumber | Broccoli^e^ | 29 ± 8 |
| 62 | Leafy vegetables | Broccoli^e^ | 29 ± 8 |
| 63 | Baked vegetables (leafy) | Broccoli^e^ | 29 ± 8 |
| 64 | Pumpkin | Broccoli^e^ | 29 ± 8 |
| 65 | Squash | Broccoli^e^ | 29 ± 8 |
| 66 | Eggplant | Broccoli^e^ | 29 ± 8 |
| 67 | Celery | Broccoli^e^ | 29 ± 8 |
| 68 | Green peas | Peas, steamed^e^ | 37 ± 8 |
| 69 | Green beans | Peas, steamed^e^ | 37 ± 8 |
| 70 | Carrot | Carrot^e^ | 44 ± 7 |
| 71 | Garlic | Broccoli^e^ | 29 ± 8 |
| 72 | Onion | Broccoli^e^ | 29 ± 8 |
| 73 | Cabbage | Cauliflower, steamede^e^ | 48 ± 9 |
| 74 | Spinach | Broccoli^e^ | 29 ± 8 |
| 75 | Turnip | Peas, steamed^e^ | 37 ± 8 |
| 76 | Beet | Carrot^e^ | 44 ± 7 |
| 77 | Pepper | Broccoli^e^ | 29 ± 8 |
| 78 | Tomato paste | Tomato pasta sauce^c^ | 41 ± 8 |
| 79 | Pickle | Coleslaw, commercial^c^ | 20 ± 2 |
| 80 | Salty cucumber | Coleslaw, commercial^c^ | 20 ± 2 |
| 81 | Melon | Melon^c^ | 93 ± 15 |
| 82 | Cantaloupe | Melon^c^ | 93 ± 15 |
| 83 | Watermelon | Melon^c^ | 93 ± 15 |
| 84 | Pear | Apple^c^ | 43 ± 3 |
| 85 | Apricot | Peach, raw^e^ | 39 ± 18 |
| 86 | Cherry | Seedless raisins^c^ | 31 ± 5 |
| 87 | Apple | Apple^c^ | 43 ± 3 |
| 89 | Peach | Peach, raw^e^ | 39 ± 18 |
| 90 | Nectarine | Peach, raw^e^ | 39 ± 18 |
| 91 | Aloche (green tomato) | Seedless raisins^c^ | 31 ± 5 |
| 92 | Fig | Seedless raisins^c^ | 31 ± 5 |
| 93 | Dried fig | Seedless raisins^c^ | 31 ± 5 |
| 94 | Grapes | Black grapes^c^ | 60 ± 4 |
| 95 | Kiwi | Orange^c^ | 44 ± 2 |
| 96 | Orange | Orange^c^ | 44 ± 2 |
| 97 | Persimmon | Apple^c^ | 43 ± 3 |
| 98 | Tangerine | Orange^c^ | 44 ± 2 |
| 99 | Pomegranate | Orange^c^ | 44 ± 2 |
| 100 | Date | Seedless raisins^c^ | 31 ± 5 |
| 101 | Plum | Peach, raw^e^ | 39 ± 18 |
| 102 | Sour cherry | Seedless raisins^c^ | 31 ± 5 |
| 103 | Strawberry | Orange^c^ | 44 ± 2 |
| 104 | Lemon | Orange^c^ | 44 ± 2 |
| 105 | Citrus | Orange^c^ | 44 ± 2 |
| 106 | Grapefruit juice | Apple juice^c^ | 47 ± 2 |
| 107 | Orange juice | Apple juice^c^ | 47 ± 2 |
| 108 | Apple juice | Apple juice^c^ | 47 ± 2 |
| 109 | Melon juice | Apple juice^c^ | 47 ± 2 |
| 110 | Margarine | Butter^c^ | 2 ± 1 |
| 111 | Pineapple compote | Apple juice^c^ | 47 ± 2 |
| 112 | Raisins | Seedless raisins^c^ | 31 ± 5 |
| 113 | Muskmelon | Melon^c^ | 93 ± 15 |
| 114 | Banana | Banana^c^ | 59 ± 4 |
| 115 | White berry | Seedless raisins^c^ | 31 ± 5 |
| 116 | Dried white berry | Seedless raisins^c^ | 31 ± 5 |
| 117 | Dried peach | Seedless raisins^c^ | 31 ± 5 |
| 118 | Dried apricot | Seedless raisins^c^ | 31 ± 5 |
| 119 | Olive | Walnut^c^ | 5 ± 1 |
| 120 | Compote | Apple juice^c^ | 47 ± 2 |
| 121 | Solid oil | Butter^c^ | 2 ± 1 |
| 122 | Liquid oil (overall) | Olive oil^d^ | 2 ± 1 |
| 123 | Olive oil | Olive oil^d^ | 2 ± 1 |
| 124 | Fat (sheep, cow) | Butter^c^ | 2 ± 1 |
| 125 | Animal fat | Butter^c^ | 2 ± 1 |
| 126 | Mayonnaise | Cream^e^ | 8 ± 8 |
| 127 | Peanut | Walnut^c^ | 5 ± 1 |
| 128 | Almond | Walnut^c^ | 5 ± 1 |
| 129 | Walnut | Walnut^c^ | 5 ± 1 |
| 130 | Pistachio | Walnut^c^ | 5 ± 1 |
| 131 | Hazelnut | Walnut^c^ | 5 ± 1 |
| 132 | Sunflower seed | Walnut^c^ | 5 ± 1 |
| 133 | Sugar loaf | Glucose^c^ | 100 |
| 134 | Sugar | Glucose^c^ | 100 |
| 135 | Honey | Raspberry jam^c^ | 62 ± 9 |
| 136 | Jam | Raspberry jam^c^ | 62 ± 9 |
| 137 | Coca-Cola | Coca-Cola^c^ | 44 ± 3 |
| 138 | Beverage | Coca-Cola^c^ | 44 ± 3 |
| 139 | Gaz (sweet) | Fat-free blueberry muffin^c^ | 69 ± 6 |
| 140 | Chocolate | Mars bar^c^ | 89 ± 11 |
| 141 | Tea | - | 0 |
| 142 | Salt | - | 0 |
| 143 | Coffee | - | 0 |
| 144 | Lemon juice | - | 0 |
| 145 | Nabat | Glucose^c^ | 100 |
| 146 | Mushroom | Broccoli^e^ | 29 ± 8 |
| 147 | Halva | Fat-free blueberry muffin^c^ | 69 ± 6 |
| 148 | Sugar-rich halva | Fat-free blueberry muffin^c^ | 69 ± 6 |
| 149 | Flour | White bread^c^ | 73 ± 5 |
| 150 | Mung bean | Baked beans^c^ | 88 ± 14 |
| 151 | Flavored milk | Milk^c^ | 24 ± 3 |
| 152 | Strained yogurt | Yoghurt^e^ | 46 ± 19 |
| 153 | Low fat yogurt | Yogurt, strawberry low-fat^c^ | 84 ± 9 |
| 154 | High fat yogurt | Yoghurt^e^ | 46 ± 19 |
| 155 | Baked carrot | Carrot^e^ | 44 ± 7 |
| 156 | Okra | Broccoli^e^ | 29 ± 8 |
| 157 | Baked onion | Broccoli^e^ | 29 ± 8 |
| 158 | Salty pickle | Coleslaw, commercial^c^ | 20 ± 2 |
| 159 | Grapefruit | Orange^c^ | 44 ± 2 |
| 160 | Pineapple | Apple^c^ | 43 ± 3 |
| 161 | Nokhodchi | Walnut^c^ | 5 ± 1 |
| 162 | Noql | Fat-free blueberry muffin^c^ | 69 ± 6 |
| 163 | Pirashki | Fat-free blueberry muffin^c^ | 69 ± 6 |
| 164 | Qottab | Fat-free blueberry muffin^c^ | 69 ± 6 |
| 165 | Baqlava | Fat-free blueberry muffin^c^ | 69 ± 6 |
| 166 | Loz | Fat-free blueberry muffin^c^ | 69 ± 6 |
| 167 | Pashmak | Fat-free blueberry muffin^c^ | 69 ± 6 |
| 168 | Haji badam | Fat-free blueberry muffin^c^ | 69 ± 6 |
| 169 | Nan berenji | Fat-free blueberry muffin^c^ | 69 ± 6 |
| 170 | Sohan | Glucose^c^ | 100 |
| 171 | Candy | Glucose^c^ | 100 |
| 172 | Caramel | Fat-free blueberry muffin^c^ | 69 ± 6 |
| 173 | Shirini khoshk | Apple pie^c^ | 47± 4 |
| 174 | Shirini tar | Apple pie^c^ | 47± 4 |
| 175 | Pofak | All-Bran Complete Wheat Flakes^c^ | 55 ± 7 |
| 176 | Abgosht | Peas, steamed^e^ | 37 ± 8 |
| 177 | Chips | Potato chips^c^ | 45 ± 10 |
| 178 | Sesame seed paste | Fat-free blueberry muffin^c^ | 69 ± 6 |

^a^ The insulin index for food items was obtained from the previous studies published by Bao et al (1), Bell et al (2), and Holt et al(3). The FII of similar food items based on the similarity between their energy, carbohydrate, fiber, fat, and protein content was applied in the current investigation for food items that were not available in the food list released by previous studies. The insulin index for 4 food items including lemon juice, tea, coffee, and salt was considered 0 because the energy, carbohydrate, protein, and fat content of these foods is close to 0.

^b^FII values are presented as mean ± standard error.

^c^ Study of Bao et. al (1)

^d^ Study of Holt et. al (3)

^e^ Study of Bell et. al (2)

**References:**

1. Bao J, de Jong V, Atkinson F, Petocz P, Brand-Miller JC. Food insulin index: physiologic basis for predicting insulin demand evoked by composite meals. The American journal of clinical nutrition. 2009;90(4):986-92.

2. Bell KJ, Petocz P, Colagiuri S, Brand-Miller JC. Algorithms to Improve the Prediction of Postprandial Insulinaemia in Response to Common Foods. Nutrients. 2016;8(4):210.

3. Holt SH, Miller JC, Petocz P. An insulin index of foods: the insulin demand generated by 1000-kJ portions of common foods. The American journal of clinical nutrition. 1997;66(5):1264-76.
